# Skeletal and Cardiac Rhabdomyolysis in a Live-Stranded Neonatal Bryde's Whale With Fetal Distress

**DOI:** 10.3389/fvets.2019.00476

**Published:** 2019-12-20

**Authors:** Nakita Câmara, Eva Sierra, Antonio Fernández, Cristian Manuel Suárez-Santana, Raquel Puig-Lozano, Manuel Arbelo, Pedro Herráez

**Affiliations:** Department of Veterinary Histology and Pathology, Veterinary School, Institute of Animal Health and Food Safety, University of Las Palmas de Gran Canaria, Las Palmas de Gran Canaria, Spain

**Keywords:** Bryde's whale (*Balaenoptera edeni*), cetaceans, rhabdomyolysis, live-stranding, *Mysticeti*, neonate, stress cardiomyopathy

## Abstract

The main objective of wildlife forensic investigation is to recognize pathologic changes and cause of death. Even though it may not always be possible to determine the specific illness and/or etiology, the description and subsequent interpretation of the injuries provide an invaluable understanding of pathology in cetacean post-mortem investigations. Although pathological studies have been previously reported in various cetacean species, such descriptions of the infraorder *Mysticeti* remain rare. A live-stranded neonatal Bryde's whale (*Balaenoptera edeni*) which subsequently died soon after the stranding, was assessed by physical exam, blood examination, gross necropsy evaluation, histopathology, and immunohistochemistry. It presented with elevated serum levels of creatine kinase, cardiac troponin I, urea, and creatinine. Microscopically, we observed keratin spicules (squamous epithelial cells) and areas of atelectasis in the lungs. Acute degeneration in the myocytes and cardiomyocytes were comparable to the findings previously described in cases of capture myopathy in live-stranded cetaceans. Immunohistochemistry biomarkers such as myoglobin, fibrinogen, and troponin were analyzed. Skeletal and myocardial damage has been documented in several cetacean species. However, this is the first reported case of skeletal and cardiac rhabdomyolysis associated with live-stranding in a newborn Bryde's whale that suffered from fetal distress.

## Background

Pathological study of wildlife fauna has the disadvantage of an unknown clinical history of the animal. The complexity is enhanced in cetaceans because of the difficulty of performing clinical exams and/or other analyses on live animals. The most feasible technique for health assessment in dead cetaceans is by detection of injuries in these animals, through pathological study. The importance of these studies is recognized worldwide to promote conservation of these animals. Unfortunately, the description of pathological entities and/or causes of death in the infraorder *Mysticeti* remains rare (1–8).

Live-stranding is a pathological state with severe acute stress and physical damage central to its etiopathogenesis. It presents clinical and traumatic findings that can cause death of the animal, or can seriously aggravate an existing condition over the period of stranding, capture, handling, restraint, transportation and/or captivity (1, 2, 9–15). The response mechanisms and resultant damage to multiple systems involved in live-stranded cetaceans are comparable to exertional rhabdomyolysis (capture myopathy) in many animals, including birds and terrestrial or marine wild mammals (1, 9–11, 14, 16). Although pathological findings may vary among individuals, biochemical changes and histopathological lesions, consisting of ischemia-reperfusion injuries, are often observed. These changes result in local-to-generalized vasospasms and vasodilation (catecholamine surge, neurogenic shock, and impeded venous flow return by body compression), which is analogous to the stress cardiomyopathy in humans and in direct traumatic injury to muscles, resulting in acute to subacute degeneration (rhabdomyolysis). Acute renal failure associated with myoglobinuric nephrosis secondary to muscle damage and areas of necrosis in viscera are also observed (1, 9, 10, 12, 16–27).

In both wild and captive cetaceans, neonatal mortality is a recognized concern (28–31). The main causes of stranding and/or death in newborns are related with problems in pregnancy (abortion, prematurity), childbirth (fetal distress, dystocia), nursing (missed transfer of passive immunity), behavior (maternal-filial separation/maternal neglect), or intra and interspecific interactions with a fatal outcome. All these above are enclosed in the category of neonatal and/or perinatal pathologies (1, 2). In the case of asphyxia, the fetus responds with redistribution of the blood flow, which limits the deleterious effects of oxygen deprivation in vital organs. This enables the fetus to survive intact unless the asphyxia is profound or prolonged (32).

This report describes the biochemical analysis and gross, histopathological, histochemical, and immunohistochemical features in a live-stranded neonatal Bryde's whale.

## Case Presentation

### Stranding Circumstances

A 393-cm-long newborn male Bryde's whale was stranded on the coast of Fuerteventura, Canary Island, Spain, in September 2016. Observations between the high and low tide revealed that the animal appeared to be alive and few meters from the coast. At low tide, the animal was stranded on the beach and died before specialized assistance could be given.

### Biochemical Analysis

A sample of whole blood was collected from the tail flukes, immediately post-mortem, for analysis of the serum. Biochemical markers of acute skeletal and heart muscle damage, creatine kinase (CK 460.0 U/L), and cardiac troponin I (0.20 μg/L), were analyzed. Kidney function was also assessed via blood urea nitrogen (BUN 162 mg/dL) and creatinine (2.4 mg/dL).

### Gross Anatomic Analysis

**Figure 1:**
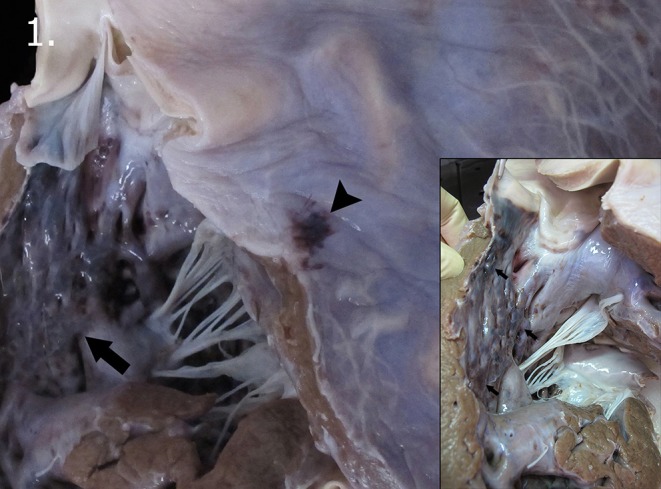
Macroscopic vascular changes observed in the heart. Subepicardial (arrow head) and subendocardial hemorrhage (arrow) in the left ventricle. Detail of the subendocardial hemorrhages present in the left ventricle (arrows).

A thorough necropsy was performed on the calf, following the standard protocol published by the European Society of Cetaceans and with the addition of some procedures detailed in the *Marine Mammals Ashore* manual, to determine the cause of death (14, 33). The animal was in poor body/nutritional condition with several linear erosions, distributed in a multifocal manner on the ventral abdomen (attributed to direct active stranding damage). While several vestigial hairs were noted in the lateral part of the maxilla, the navel was not healed and contained an internal white exudate. During dissection of the subcutaneous planes, moderate diffuse hemorrhages were observed, especially in the ventral region. The muscles were pale yellow-to-orange. The epiglottis was flaccid at the rostral level, the trachea showed mild-to-moderate foam, and the main and secondary bronchi and bronchioles presented abundant foam, representing pulmonary edema. Both lungs had multifocal and local extensive areas of dark reddish color and were firm (compatible with pulmonary atelectasis). Mild-to-moderate exudation of blood was noted at the incision. Some serous fluid was detected in the pericardial space. On sectioning, both ventricles presented moderate-to-severe subepicardial and subendocardial hemorrhage (Figure 1). The ductus arteriosus was also present. Moderate and diffuse mucosal congestion was observed in the stomach, liver, bladder, sclera, and meningeal and subarachnoid vessels. The cerebellum displayed moderate and diffuse congestion, edema, and mild-to-moderate hemorrhage.

### Histopathological Analysis

Representative tissue samples were fixed in 10% formalin for ~48 h and processed using standard protocol. The skeletal (*longissimus dorsi* and *rectus abdominis*) and heart muscles (both atria and ventricles), atrioventricular valves (bicuspid or mitral and tricuspid), semilunar valves (sigmoid, aortic, and pulmonary with the corresponding arteries), and kidneys were examined for the potential presence of rhabdomyolysis and myoglobinuric nephrosis. Tissue sections (4-μm-thick) were used for hematoxylin and eosin and periodic Acid-Schiff staining, while 5-μm-thick layers were used for phosphotungstic acid, hematoxylin, and Masson's trichrome techniques.

**Figure 2:**
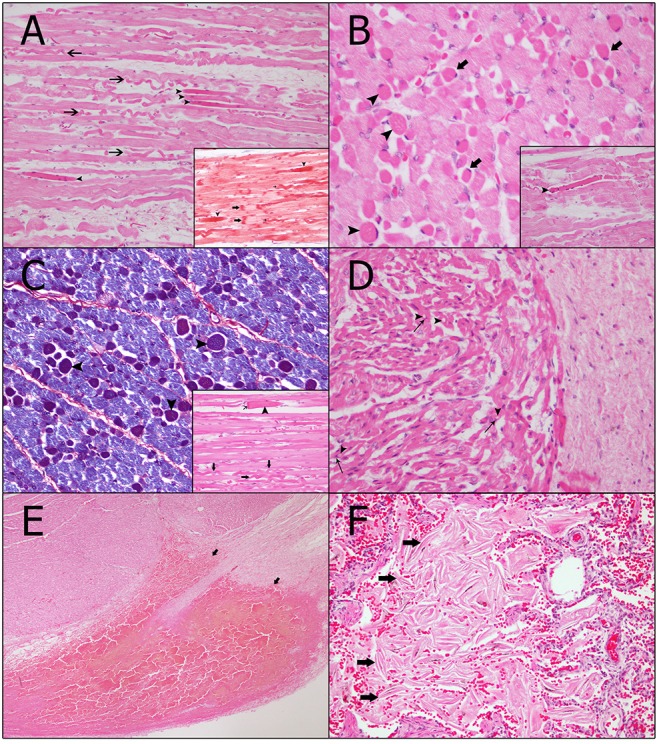
Vascular and acute degenerative changes observed in the skeletal and heart muscles. **(A)** Long and thin undulated fibers, also referred to as wavy fibers (arrows), can be seen in the myocytes. In addition, the myocytes demonstrate hypereosinophilia, i.e., an increase in staining of necrotic muscular cells (arrow heads) with different histochemical techniques, which is usually associated with segmental hypercontraction (arrow heads) (hematoxylin and eosin technique, magnification: 40×). Inset: The contraction band necrosis (arrows) runs transversely throughout the myocytes and is identified via the increasing red color intensity (Masson's trichrome technique). Furthermore, both hypereosinophilia and wavy fibers can be observed (thin arrows) (magnification: 40×). **(B)** In the transversal cut, myocytes of minor caliber (most likely type I fibers) are affected and present hypereosinophilia with segmental hypercontraction (arrow heads). Moreover, endomysia edema (thin arrows) can be seen (hematoxylin and eosin technique, magnification: 60×). Inset: Detail of the segmental hypercontraction of a myocyte (hematoxylin and eosin technique, magnification: 40×). **(C)** More intense blue coloring of the damaged myocytes of minor caliber, which identifies hypereosinophilia and segmental hypercontraction (arrow heads) (phosphotungstic acid hematoxylin technique, magnification: 40×). Inset: Detail of a myocyte with segmental hypercontraction (arrow head) and segmental necrosis of the fiber with the retraction cap (thin arrow). Wavy fibers can also be observed (arrows) (hematoxylin and eosin technique, magnification: 40×). **(D)** Cardiomyocytes show vacuolar degeneration (arrow heads) and pyknotic nucleus (thin arrows) (hematoxylin and eosin technique, magnification: 40×). **(E)** Vascular changes present in the heart consistent with a subepicardial hemorrhage in the left ventricle (arrows) (hematoxylin and eosin technique, magnification: 4×). **(F)** Detail of the intra-alveolar keratin spicules (arrows) (hematoxylin and eosin technique, magnification: 40×).

On histopathologic and histochemical examinations, the skeletal and heart muscles presented with injuries consistent with vascular changes (i.e., hemorrhages and interstitial edema) and acute degenerative lesions (i.e., contraction band necrosis, wavy fibers, segmental hypercontraction, hypereosinophilia, cytoplasmic vacuolization, and nuclear pyknosis). Contraction band necrosis was observed in the *longissimus dorsi* and *rectus abdominis* (Figure 2A). Long and thin undulated fibers (wavy fibers) were noted (Figure 2A). Hypereosinophilia (Figures 2B,C) was usually associated with either segmental hypercontraction or segmental necrosis. The above-mentioned changes in the skeletal muscle were moderate and illustrated a multifocal pattern with smaller diameter fibers (presumably type I fibers). Atria and ventricles displayed a multifocal, moderate-to-severe degree of interstitial edema, wavy fibers, hypereosinophilia, and cytoplasmic vacuolization with pyknotic nucleus (Figure 2D). Both ventricles demonstrated mild-to-moderate, multifocal, subepicardial, and subendocardial hemorrhage (Figure 2E).

Additional histopathological findings include (a) discrete capsular hemorrhage and mild-to-moderate, diffuse congestion in the kidneys with (b) mild, multifocal dilatation of the renal tubules; (c) in the lungs, severe, multifocal presence of keratin spicules (squamous epithelial cells) in alveolar spaces (Figure 2F) and (d) severe, multifocal, local extensive areas of atelectasis and moderate, multifocal alveolar hemorrhages; (e) severe, diffuse macro and microvacuolar degeneration (hyaline globules) in the hepatocytes; (f) moderate, focal suppurative omphalitis with the presence of coccoid bacterial colonies in the most superficial areas of the navel, (g) mild congestion and hemorrhage in multiple organs.

### Immunohistological Analysis

Tissue sections (3 μm thick) were immunolabeled with anti-myoglobin (skeletal and cardiac muscles, and the kidneys), anti-fibrinogen (skeletal and cardiac muscles), anti-cardiac troponin I (cardiac muscle), and anti-cardiac troponin C (cardiac muscle) primary antibodies. They were visualized using the VECTASTAIN® Elite ABC-Peroxidase Kit (PK-6100) from Vector Laboratories (Peterborough, United Kingdom). The immunohistochemical methodology is summarized in Supplementary Table 1. The negative control for the latter consisted of serial sections of the heart without the primary antibody. In contrast, the positive control for myoglobin and fibrinogen were from a cetacean heart sample of a striped dolphin (*Stenella coeruleoalba*). The dolphin had been stranded alive and developed CM owing to capture and human interaction during the rehabilitation process (9, 10). Heart samples from a pig and cetacean, with no apparent acute macroscopic and/or histological lesions, were used as positive controls for cardiac troponin I and cardiac troponin C.

**Figure 3:**
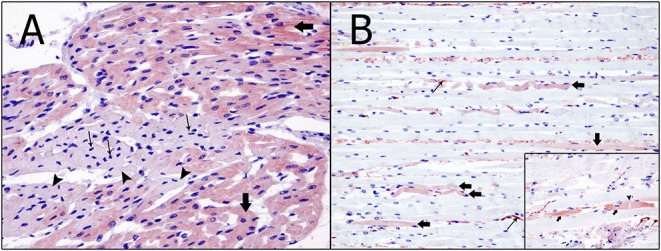
Immunohistochemical techniques in skeletal and heart muscles. **(A)** Degenerated/necrotic cardiomyocytes (arrow heads), with pyknotic nucleus (thin arrows), show intrafibrillar depletion of cardiac troponin C. In contrast, normal cardiomyocytes (arrows) present an intense immunolabeling (immunohistochemical technique: anti-troponin C, magnification: 60×). **(B)** Expression of fibrinogen (arrows) in the myocytes presenting changes, including wavy fibers, hypereosinophilia and segmental hypercontraction. Immunolabeling of fibrinogen in the interior of the blood vessels can also be seen (thin arrows) (immunohistochemical technique: anti-fibrinogen, magnification: 40×). Inset: Necrotic myocytes (arrows) strongly expressed alongside the contraction band necrosis (arrow head) and inside the blood vessels (thin arrows) (immunohistochemical technique: anti-fibrinogen, magnification: 40×).

Immunohistochemically, the degenerated/necrotic muscular and heart cells showed homogenous, intrafibrillar depletion of cardiac troponin I, cardiac troponin C (Figure 3A), and myoglobin. Damaged cells from the skeletal and cardiac muscles were found to exhibit several concentrations of immunolabeling for fibrinogen (Figure 3B). The kidneys did not exhibit any accumulation of myoglobin.

## Discussion

### Discussion of the Animal Characteristics

Cetacean newborns/neonates are defined as having a compatible total length, displaying “fetal folds” over the body, soft and folded dorsal fin and tail flukes, vibrissal hairs or vibrissal crypts, and a healing (or closing) navel (34). Our animal was 393 cm long, slightly under the normal range (395–430 cm) (35). The animal did not present any “fetal folds” but we identified some vibrissal hairs; the navel was not healed and contained inflammatory exudate. This suggested an infection after birth; therefore, the animal would most likely be a few days old (34).

### Discussion of the Biochemical Results

Clinico-pathological evaluation was challenging in this case as biochemical values, such as that for cardiac troponin I, are rarely reported. They may not exist in the scientific database for various species of cetaceans, particularly the infraorder *Mysticeti*. Our biochemical data was compared with published papers assessing different mammals, including humans, dogs, and other species of cetaceans [e.g., bottlenose dolphins (*Tursiops truncatus*) belonging to the infraorder *Odontoceti* and a stranded baby gray whale (*Eschrichtius robustus*) of the infraorder *Mysticeti*]; this is summarized in Table 1 (13, 36–39).

**Table 1:** Comparison between the biochemical results of the studied animal and normal laboratory values of other mammals.

	**Class** ***Mammalia***					
	**Order *Primates***	**Order *Carnivora***	**Order** ***Cetartiodactyla***			
	**Suborder *Haplorhini***		**Suborder** ***Cetacea***			
	**Infraorder *Simiformes***		**Infraorder** ***Odontoceti***		**Infraorder** ***Mysticeti***	
	**Family *Hominidae***	**Family *Canidae***	**Family** ***Delphinidae***		**Family *Eschrichtiidae***	**Family *Balaenopteridae***
	**HUMANS (*Homo sapiens*) (17)**	**DOG (*Canis lupus familiaris*) (19, 20)**	**BOTTLENOSE DOLPHIN (*****Tursiops truncatus*****) (****9****)**		**NEONATAL GRAY WHALE (*Eschrichtius robustus*) (9)**	**NEONATAL BRYDE'S WHALE (*Balaenoptera edeni*)**
			**Captive (*n* = 38)**	**Wild Atlantic Juvenile (*n* = 96)**	**Stranded (*n* = 1)**	**Stranded (n = 1)**
Creatine Kinase (U/L)	30–170	0–190	100–250	47–455	107–255	**460**
Troponin I (μg/L)	≤0.1	≤0.03–0.07	ND	ND	ND	**0.20**
Blood Urea Nitrogen (mg/dL)	8–20	7–20.72	42–58	42–77	21–75	**162**
Creatinine (mg/dL)	0.7–1.3	0.44–1.595	1.0–2.0	0.68–1.49	1.0–2.0	**2.4**

In concordance with the results obtained from other species (i.e., 107–255 U/L in a neonatal gray whale), injury to the skeletal muscle was supported by an increase in CK (460 U/L), which is one of the useful indicators of both skeletal and cardiac muscle damage (12, 13, 16, 39–41). After myocardial injury, CK begins to rise in 4 to 9 h, peaks at 24 h, and returns to baseline 48 to 72 h after the stress event (42). This increase in CK was correlated with histological and immunohistochemical changes, thus supporting the presence of muscle damage.

Cardiac troponin I, measured by conventional assays, is elevated in >90% of patients with stress cardiomyopathy (43). Our animal presented with 0.20 μg/L cardiac troponin I in the serum; this is higher than the reference values in both humans (≤ 0.1 μg/L) and dogs (≤ 0.03–0.07 μg/L) (36–38). The release of troponin from injured cardiomyocytes usually occurs 3 to 9 h after ischemic damage, peaks in 12 to 48 h, and remains elevated for 4 to 7 days (42, 44, 45). Hence, the above laboratory values cannot aid in early detection of myocardial necrosis (1–3 h). These markers do not assist in accurate diagnosis until 6 or more hours after the onset of the event. In order to obtain a satisfactory clinical picture in humans, blood should be drawn 6–9 h after the onset of the stress event and/or symptom onset (44). The increase in cardiac troponin I serum levels and the decrease in myocyte troponin immunoreaction is caused by the early release of cardiac troponin I and troponin C by damaged cardiomyocytes. This was verified by immunohistochemistry (22, 46).

In this case, pre-renal azotemia is likely related to hypovolemic shock, and was reasonably supported by higher BUN values (162 mg/dL) than the reference values from the other species (i.e., 21 to 75 mg/dL in the stranded gray whale). Hypovolemic shock can be originated by a relative decrease in the effective circulating volume without a loss of total body fluid (i.e., decreased in venous return) and/or a direct intravascular fluid loss (i.e., dehydration or hemorrhage) (12, 40). In our case, we can associate this clinical finding with various causes, such as compartment syndrome, heart failure, and dehydration. Moreover, creatinine was slightly above (2.4 mg/dL) the normal values seen in other species (i.e., 1.0 to 2.0 mg/dL in a stranded neonatal gray whale). In order to confirm the hypothesis that the increase in creatinine we observed could be within the normal ranges for the Bryde's whale species, more accurate age and species-specific normal values would be needed. Small elevation of creatinine and high levels of urea can be associated with pre-renal azotemia caused by dehydration. This is a possible interpretation for our case. Since dehydration is associated with prolonged fasting, it is important to consider this aspect in stranded animals, especially young ones (47).

### Discussion of the Histopathological Results

With the anatomopathological findings, the morphological diagnoses include (1) severe multifocal fetal atelectasis with presence of severe multifocal keratin spicules and a moderate alveolar hemorrhage; (2) multifocal moderate-to-severe acute degeneration of cardiomyocytes; (3) moderate multifocal acute skeletal muscle degeneration; and (4) mild congestion and hemorrhage in various organs.

Atelectasis is a relatively frequent finding in fetal and neonatal (*atelectasis neonatorum*) deaths (perhaps associated with aspiration of amniotic fluid or meconium), it is found incidentally or in non-specific forms in young or adult individuals (1, 48–50). In contrast, pulmonary edema with intra-alveolar keratin spicules (*pulmonary vernix caseosa*), either isolated or in aggregates of stratified epithelium, keratinized and with nuclear retention, can be observed in fetal distress. Considering non-specific findings, without knowledge of the primary cause, and after discarding lesions compatible with other etiological diagnoses, we considered the macro and microscopic findings as a whole to be typical of fetal distress (1).

Acute degenerative changes, such as contraction band necrosis, were observed in both the *longissimus dorsi* and the *rectus abdominis*. This represents a skeletal and myocardial lesion characteristic of transient ischemia and reperfusion, which is associated with high concentrations of endogenous catecholamines (23, 51, 52). This condition has been reported in humans after stressful events, as well as in other animals after acute death, including seals and cetaceans (1, 9–12, 16–23, 53–56). Wavy fibers were also detected in the skeletal and heart muscles. Considering that they are the first histologic abnormality associated with ischemia, this condition may be used as a morphological indicator of early myocardial injury (9–11, 22, 23, 53, 57). In addition, hypereosinophilia was observed in the skeletal and cardiac muscles, respectively. Animals that die following a stressful situation present with this cytoplasmic alteration (9–11, 22, 53, 57, 58). The animal also presented with vacuolization in the cardiomyocytes. Previous studies commonly associate this with areas that experience severe, chronic, and fatal ischemia as a result of acute death due to stressful situations (11, 22, 53, 59).

Vascular changes, including congestion, interstitial edema, and hemorrhage, are generally detected through histological approaches and form part of the stress cardiomyopathy pathology (17). In the current case, all the heart sections showed separated fibers, with interstitial edema. Following the introduction of catecholamines, interstitial edema is usually associated with subendocardial and subepicardial hemorrhage, found in both ventricles of this neonate (57). These lesions are occasionally detected in humans with stress cardiomyopathy and have been previously demonstrated in animals that died after live-stranding and handling (1, 9, 10, 17, 20).

The sequence of changes in an acute ischemic injury begins within 5 min. The myocardium reveals long, thinned, wavy fibers separated by spaces, characterizing edema and microvascular congestion at the borders of the ischemic myocardium. In 2 to 3 h, early changes of cardiomyocyte coagulation necrosis with nuclear pyknosis, color change, more specifically “brick red change” or cytoplasm hypereosinophilia, focal contraction bands, and subtle interstitial edema are evident. Hypereosinophilia and edema become more pronounced and more easily recognizable 3 to 6 h after the event. Six to 12 h later changes accelerate and more extensive contraction band necrosis with reperfusion is noted (25). Based on the acute degenerative findings in both skeletal and cardiac muscle of our case, we propose that the ischemic injury, which caused these lesions, occurred between 6 and 12 h prior to death, coinciding with the live-stranding.

### Discussion of the Immunohistochemical Results

Previous studies demonstrated the necessity of corroborating histopathological findings with specific markers to better determine the amount of damage present in cells. The immunohistochemical confirmation ante-mortem showed depletion of myoglobin, cardiac troponin I, and cardiac troponin C as well as intrafibrillar fibrinogen deposition (9, 10, 12, 22, 45, 58, 60–63). Depletion of myoglobin (a marker used for skeletal and cardiac damage), troponin I and C (specific markers to detect injury to the heart), as well as accumulation of fibrinogen (used to identify skeletal and cardiac damage) in injured cells was confirmed in the present study.

Although this animal presented with clinical and pathological findings resembling rhabdomyolysis, which can lead to a secondary myoglobinuric nephrosis, lesions associated with acute kidney injury (i.e., intrinsic kidney disease/ damage or acute tubular necrosis) were not detected through the histopathological and immunohistochemical studies (1, 9, 10, 12, 16–27, 64).

### Discussion of the Cause of Death

Considering the biochemical results and the macro and microscopic findings which concur with the etiological diagnoses of fetal distress and skeletal and cardiac rhabdomyolysis, we propose that the most probable cause of death in this animal is active stranding pathology which aggravated a previous neonatal/perinatal pathology.

The active stranding pathology is defined by a set of lesions and biochemical findings in animals that were stranded alive and leads to both a catecholaminergic crisis (stress cardiomyopathy) and multi-organ ischemic-reperfusion damage with rhabdomyolysis with myoglobinuric nephrosis secondary to muscle damage. The severity of this syndrome usually causes the death of the animal, occasionally as a result of the intensification of preexisting pathologies (1, 9, 10, 22).

Neonatal/perinatal pathology in cetaceans comprises a wide constellation of etiologic factors, including fetal distress (1, 2). A severe disturbance in the oxygen supply to the fetus can have effects on the newborn's cardiac function. Elevated levels of cardiac troponin I, cardiac troponin T, CK and its fraction MB can be observed in full-term infants after intrauterine hypoxia and respiratory distress (65). Limited studies have shown premature infants and various breeds of stillborn cow calves to present with acute degenerative changes, such as myocardial necrosis, which may result from antepartum or intrapartum asphyxia (hypoxia) (65–67).

## Conclusions

Although we cannot confirm that the elevated serum values (CK, cTnI, BUN, and creatinine) were due to post stranding or fetal distress, but based on the histological findings, we can conclude that these lesions are due to live stranding. Therefore, we suggest that the animal assessed here probably died because of an exacerbation of preceding injuries (fetal distress) and the final complications of stranding. Since description of pathological entities and/or causes of death in the *Mysticeti* infraorder is still scarce, we consider this article to be an important contribution to improve conservation efforts by reducing the mortality of these animals.

## Data Availability Statement

All data reported in this work is classified and stored in the tissue bank of the Institute of Animal Health and Food Safety (IUSA), Veterinary School, University of Las Palmas de Gran Canaria (ULPGC).

## Ethics Statement

Ethical review and approval was not required for the animal study because no experiments were performed on live animals. Required permission for the management of stranded cetaceans was issued by the environmental department of the Canary Islands' Government and the Spanish Ministry of Environment. No experiments were performed on live animals.

## Author Contributions

NC wrote the article, collected the blood sample, performed the necropsy of the animal, and contributed toward the biochemical analysis, gross, histological, histochemical, and immunohistological description and diagnosis of the case. ES and PH contributed toward the biochemical analysis, gross, histological, histochemical, and immunohistological description and diagnosis of the case, and guided NC during the drafting and publication processes. AF, CS-S, RP-L, and MA performed the necropsy of the animal and contributed toward the gross and histological description and diagnosis of the case. All authors read and approved the final manuscript.

### Conflict of Interest

The authors declare that the research was conducted in the absence of any commercial or financial relationships that could be construed as a potential conflict of interest.
